# Bats reveal the true power of influenza A virus adaptability

**DOI:** 10.1371/journal.ppat.1008384

**Published:** 2020-04-16

**Authors:** Kevin Ciminski, Florian Pfaff, Martin Beer, Martin Schwemmle

**Affiliations:** 1 Institute of Virology, Medical Center–University of Freiburg, Freiburg, Germany; 2 Faculty of Medicine, University of Freiburg, Freiburg, Germany; 3 Institute of Diagnostic Virology, Friedrich-Loeffler-Institut, Greifswald, Germany; University of Wisconsin Madison, UNITED STATES

## Introduction

Influenza A viruses (IAVs) circulate among a wide variety of different hosts. They can cross species barriers and establish new virus lineages in avian and mammalian species. As a consequence, IAVs are exposed to recurrent selective pressures, leading to a range of virus variants that are able to cope with the new host environment [1]. Bats were not considered to be part of this IAV habitat until recently, when two phylogenetically distinct IAV lineages, designated H17N10 and H18N11, were identified in the New World bats *Sturnira lilium* and *Artibeus* spp, respectively [2–4]. Likewise, Old World bats can harbor influenza viruses, as exemplified by the genetically distinct H9N2 virus found in Egyptian fruit bats (*Rousettus aegyptiacus*) [5]. The discovery of influenza viruses in New and Old World bats did severely challenge our previous conception of IAV host range and phylogeny. It, moreover, provided unexpected insights into the remarkable adaptive potential of these viruses and their evolutionary origin.

## What was first: IAVs of New or Old World bats?

**Fig 1:**
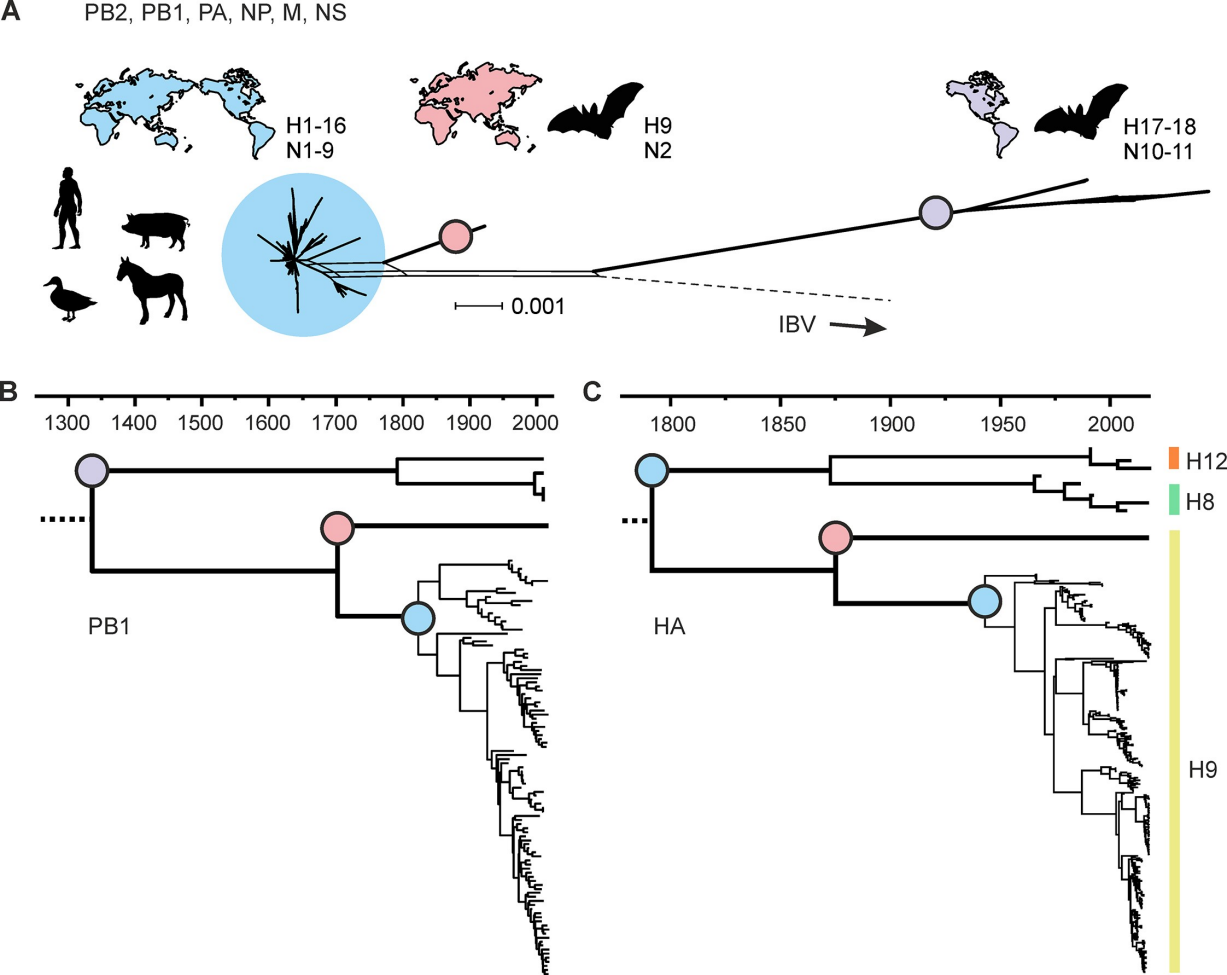
Phylogenetic analysis of the internal gene segments of conventional and bat-derived IAVs highlights the large genetic divergence and recent reassortment events. (A) The phylogenetic relationship of conventional and bat-derived IAVs was computed in a SuperNetwork [28] by using the nucleotide sequences of the internal gene segments (PB2, PB1, PA, NP, M, and NS) of 110 representative IAVs and six IBVs. All conventional non-bat IAVs (blue) are of common origin and cluster tightly. In contrast, the internal gene segments of the bat-derived IAVs form two outgroups that are located at a more basal position. Notably, Old World (red) and New World bat IAVs (purple) are widely separated. The parallel lines indicate uncertainties between the phylogenetic trees that make up the presented phylogenetic network. (B) A time-calibrated phylogeny was calculated for PB1 as a representative IAV internal gene segment. The timeline (presented in CE) shows that the New World bat IAV segments branched off more than 650 years ago (purple node) and that the last common ancestor of Old World bat IAVs (red node) and conventional IAVs (blue node) is around 300 years old. (C) Surprisingly, a comparable time-calibrated phylogeny of the Old World bat H9 HA (red node) along with conventional H8, H9, and H12 HAs (blue nodes) points to a much more recent common ancestor for the Old World bat–derived HA. See supporting information for a detailed description of the performed phylogenetic analysis (S1 Technical Appendix) and a full-sized version of the phylogenetic network (S1 Fig). HA, hemagglutinin; IAV, influenza A virus; IBV, influenza B virus; NP, nucleoprotein.

The genetic divergence of IAVs circulating in New and Old World bat species is much broader than initially anticipated. Bat-derived IAVs have a higher overall genetic diversity than conventional non-bat IAVs [3, 5]. Internal gene segments that are usually highly conserved among IAVs cluster as one narrow clade on a phylogenetic branch when non-bat IAVs are analyzed, whereas the corresponding gene segments of New and Old World bat IAVs form distant outgroups [3, 5] (Fig 1A and S1 Fig). New and Old World bat IAVs did most likely split into two genetic branches as a result of either geographic separation or multiple early spillover events of ancient viruses. Phylogenetic backdating of the internal gene segments suggests that the precursor of the New World bat IAVs separated from all other lineages more than 650 years ago, although some uncertainties commonly associated with molecular clock analyses exist (Fig 1B). Interestingly, although the Old World bat H9N2 subtype arose from a common ancestor dated around 1700 CE (Fig 1B), its H9 hemagglutinin (HA) sequence is phylogenetically similar to younger sequences isolated from avian hosts (Fig 1C). This disparity is best explained by the occurrence of more recent reassortment events between Old World bat ancestral viruses and avian strains. Furthermore, our phylogenetic analyses reveal that the newly discovered bat IAVs share a common ancestor with non-bat IAVs (Fig 1A and 1B). It is therefore conceivable that all present-day IAVs originate from bats. It is equally possible, however, that an even older IAV precursor was circulating in avian species and was later introduced into mammals—including bats.

## What is the biological role of the bat IAV surface glycoproteins?

**Fig 2:**
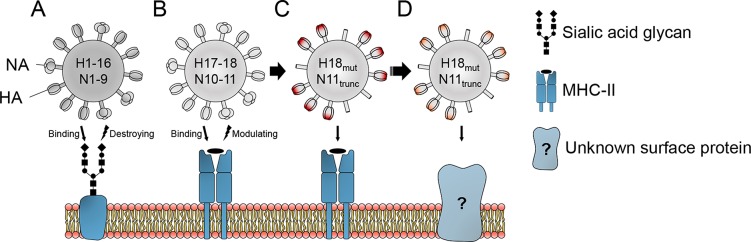
Model of the receptor binding and modulating activity of the known IAV surface glycoproteins. (A) Infection of a host cell is initiated by binding of HA subtypes H1–16 to sialic acid residues exposed on the host cell surface. These glycan structures are subsequently cleaved off by NA of the subtypes N1–9 in order to facilitate the release of viral particles. (B) The H17 and H18 HA proteins of New World bat IAVs utilize MHC-II molecules for cell entry. Preliminary data suggest that the New World bat IAV N11 NA protein decreases MHC-II surface expression by a yet unknown mechanism, allowing unhindered release of budding particles. (C) The New World bat IAV subtype H18N11 exhibits an unforeseen high flexibility to quickly acquire H18_mut_ that compensate for an N11_trunc_ and restore efficient growth in cell culture and mice. (D) This inherent flexibility of H18 might have the potential to allow further adaptations to new cell surface receptors. H18_mut_, mutations in H18; HA, hemagglutinin; IAV, influenza A virus; MHC-II, major histocompatibility complex class II; N11_trunc_, truncated N11; NA, neuraminidase.

IAVs initiate infection via the viral HA surface glycoprotein that binds to sialic acid receptors on host cell glycoproteins (Fig 2A). Until recently, it was thought that sialic acids serve as the universal receptors for all influenza virus strains, thereby facilitating cross-species transmission. Indeed, the HA of the newly discovered Old World bat H9N2 virus binds to α2,3-sialic acid moieties [5]. Surprisingly, however, H17 and H18 of the New World bat influenza strains were found to lack this property and cannot use sialic acid receptors for infection [3, 6, 7]. Instead, both New World bat–derived HA subtypes utilize major histocompatibility complex class II (MHC-II) molecules for cell entry [8, 9]. Importantly, MHC-II proteins of multiple species, including chicken, pigs, mice, and humans, can serve as receptors, indicating that receptor usage by bat IAVs does not provide a tight species barrier but is compatible with a broad host range (Fig 2B) [10]. MHC-II molecules are normally found on immune cells of the lymphoid tissue, such as B cells, macrophages, and dendritic cells [11], but they can also be expressed on epithelial cells [12]. Although MHC-II molecules are indispensable for H17- and H18-mediated cell entry [8, 9], it is presently not clear whether they serve as bona fide binding receptors or else as necessary entry cofactors. Physical interactions between H17/H18 and MHC-II molecules need to be demonstrated, and the binding interface(s) need to be defined.

Conventional IAVs and the bat H9N2 virus possess the surface glycoprotein neuraminidase (NA), which removes sialic acid residues from infected cells to facilitate the release of newly formed viral particles (Fig 2A). The New World bat–derived IAVs also encode an NA protein which, however, has no detectable sialidase activity [2, 3, 13, 14]. Its real function has remained enigmatic until recently. Preliminary data now suggest that bat N11 down-regulates the surface expression of MHC-II molecules by an as yet unknown mechanism [15] (Fig 2B). If confirmed, these data demonstrate that the surface glycoproteins of New World bat IAVs have both receptor binding and destroying activities and hence serve the same function as the HA and NA proteins of conventional IAVs.

## Why did the New World bat influenza viruses evolve this way and what are the consequences?

A well-known characteristic of IAVs is their capacity to rapidly evolve and adapt to new environments. Their adaptability is based mainly on (1) gene reassortment (i.e., the ability to exchange gene segments between strains) and (2) genetic drift that generates a vast number of viral quasispecies due to the infidelity of the viral polymerase [16]. Reassortment between two different yet compatible viruses may generate antigenically novel strains with pandemic potential [17]. Favorable mutations acquired by genetic drift allow evasion from host immune responses and promote successful adaptation to new hosts. For instance, specific amino acid substitutions in HA (E190D/G225D and Q226L/G228S) enable avian IAVs to bind and infect human cells [18]. Interestingly, as the H17 and H18 proteins of New World bat IAVs are homologs of the HA glycoproteins of conventional IAVs, they must have undergone drastic changes to accommodate MHC-II molecules as entry factors. An exchange of key amino acids in the putative receptor binding site prevents any interaction with sialic acid residues [2, 3, 6]. The underlying selection pressure that resulted in the switch of receptor usage is not known and remains a matter of speculation. A plausible scenario would be that the precursor of the presently known IAVs harbored a HA protein with a dual affinity for sialic acids and for an unknown—possibly MHC-II-related—cellular surface protein, allowing infection of both birds and bats. Possibly because of ecological separation of this ancestral influenza virus in New World bats, subsequent evolution in the new host led to increased affinity for MHC-II. Simultaneously, the evolutionary process fostered additional species-specific adaptations. These comprise for example the co-selection of genome packaging signals in conjunction with a specific set (code) of amino acids in the viral nucleoprotein (NP) [19–21]. Both the segment-specific packaging signals and a compatible NP amino acid code are compulsory to orchestrate the incorporation of the viral genome into progeny virions [22]. Therefore, because of nonmatching packaging sequences and a different NP amino acid code, New World bat and conventional non-bat IAVs are unable to reassort their genomes [20]—an additional unique feature of New World bat IAVs, making them a truly divergent entity of IAVs.

## Do New World bat IAV HAs provide a playground for future evolution?

Conventional sialic acid–binding HA and NA proteins are well studied and known to evolve rapidly in face of a rigorous host immune response [1]. The surface glycoproteins of the New World bat IAVs are still poorly understood and their adaptive potential is presently unknown. First studies show that serial passages of the H18N11 subtype in non-bat epithelial cells resulted in the emergence of efficiently replicating mutant viruses. Surprisingly, the selected mutants harbored a truncated nonfunctional N11 protein yet were able to replicate in mice, ferrets, and bats [15] (Fig 2C). Sequence analysis showed that these virus variants had acquired at least two mutations in the H18 head domain that appeared to compensate for the loss of a functional N11. Perhaps these acquired mutations reduced the binding affinity of H18 to MHC-II and supported N11-independet replication. Virus release may have been further facilitated by the fact that New World bat IAV particles bud at the apical membrane of epithelial cells [23], where MHC-II molecules are scarce [24]. Remarkably, H17N10 and H18N11 are the first IAV subtypes that utilize a proteinaceous entry receptor. The amazing ability of H18 to rapidly overcome the absence of a functional N11 suggests that the structure of H18 (and possibly H17) may provide a broader scope of evolutionary flexibility than that of conventional sialic acid–dependent IAVs. It is therefore tempting to speculate that bat IAV HA proteins, and in particular H18, might have the potential to adapt to novel and so far unknown entry receptors different from MHC-II (Fig 2D).

## How likely is a spillover of bat IAVs into the human population?

Up to now, more than 200 different viruses have been isolated from or detected in bats. Some of these bat-borne viruses, such as rabies, Ebola, severe acute respiratory syndrome (SARS), and Middle East respiratory syndrome (MERS) virus, have caused human and animal diseases, highlighting their zoonotic and epizootic threat [25]. The risk for zoonotic transmission and the associated pandemic potential that emanates from an influenza virus considerably depends on its degree of human preadaptation and the ability to overcome host restrictions. Whereas avian IAVs carry gene segments with species-specific determinants that allow efficient replication in avian but not necessarily human cells [18], the New and Old World bat IAVs appear to possess a human-compatible gene signature due to their mammalian origin. Indeed, upon serial passaging of chimeric New World bat IAVs in eggs and avian cells, these viruses readily acquired mutations in several genes. In sharp contrast, no such mutations occurred in primary human airway epithelial cell cultures [20], indicating that the internal viral proteins are well suited for the replication in human cells. Moreover, New World bat IAVs might well have the potential to infect humans, because their HAs can utilize the human MHC-II homolog human leukocyte antigen-DR isotype (HLA-DR) for cell entry [8, 9]. Nevertheless, H18N11 viruses did not induce any pathogenicity in and were not transmissible among ferrets [15], which are considered to be the best animal model for human influenza [26]. Aside from this, H18N11 is also not able to overcome the intracellular restriction imposed by major host defense factors such as the human type I and III interferon-induced antiviral factor MX (in humans, MxA), because of the lack of MxA escape mutations in NP [27]. As to bat H9N2, the usage of α2,3- instead of α2,6-sialic acid residues, together with the lack of obvious MxA resistance mutations in NP [5], suggests that the Old World bat virus possesses a low degree of human preadaptation. Thus, based on the currently available data, there is a clear, albeit low, risk for zoonotic spillover of the different bat IAVs. In view of their human-adapted internal gene segments and the ability to rapidly acquire new mutations, future transmissions to humans cannot be excluded, and proper surveillance of bat IAV distribution is indicated.

## Supporting information

S1 Fig Phylogenetic relationship of IAVs and influenza B viruses. The presented phylogenetic network is based on the segments PB2, PB1, PA, NP, M, and NS from 110 representative IAVs (highlighted in green) and six influenza B viruses (highlighted in yellow). A more detailed close-up zoom of IAVs is presented in the main text (Fig 1A), and methods are described in detail in S1 Technical Appendix. IAV, influenza A virus; NP, nucleoprotein.
(TIFF)

S1 Technical Appendix Technical information and methods used for the phylogenetic analysis. (DOCX)

## References

[1] Gorman OT, Bean WJ, Webster RG. Evolutionary processes in influenza viruses: divergence, rapid evolution, and stasis. Curr Top Microbiol Immunol. 1992;176:75–97. 10.1007/978-3-642-77011-1_6 . 1600756

[2] Tong S, Li Y, Rivailler P, Conrardy C, Castillo DA, Chen LM, et al A distinct lineage of influenza A virus from bats. Proc Natl Acad Sci U S A. 2012;109(11):4269–74. 10.1073/pnas.1116200109 22371588 PMC3306675

[3] Tong S, Zhu X, Li Y, Shi M, Zhang J, Bourgeois M, et al New world bats harbor diverse influenza A viruses. PLoS Pathog. 2013;9(10):e1003657 10.1371/journal.ppat.1003657 24130481 PMC3794996

[4] Campos ACA, Goes LGB, Moreira-Soto A, de Carvalho C, Ambar G, Sander AL, et al Bat Influenza A(HL18NL11) Virus in Fruit Bats, Brazil. Emerg Infect Dis. 2019;25(2):333–7. 10.3201/eid2502.181246 30666923 PMC6346480

[5] Kandeil A, Gomaa MR, Shehata MM, El Taweel AN, Mahmoud SH, Bagato O, et al Isolation and Characterization of a Distinct Influenza A Virus from Egyptian Bats. J Virol. 2019;93(2). 10.1128/JVI.01059-18 30381492 PMC6321940

[6] Sun X, Shi Y, Lu X, He J, Gao F, Yan J, et al Bat-derived influenza hemagglutinin H17 does not bind canonical avian or human receptors and most likely uses a unique entry mechanism. Cell Rep. 2013;3(3):769–78. 10.1016/j.celrep.2013.01.025 . 23434510

[7] Zhu X, Yu W, McBride R, Li Y, Chen LM, Donis RO, et al Hemagglutinin homologue from H17N10 bat influenza virus exhibits divergent receptor-binding and pH-dependent fusion activities. Proc Natl Acad Sci U S A. 2013;110(4):1458–63. 10.1073/pnas.1218509110 23297216 PMC3557073

[8] Giotis ES, Carnell G, Young EF, Ghanny S, Soteropoulos P, Wang LF, et al Entry of the bat influenza H17N10 virus into mammalian cells is enabled by the MHC class II HLA-DR receptor. Nat Microbiol. 2019 10.1038/s41564-019-0517-3 . 31358984

[9] Karakus U, Thamamongood T, Ciminski K, Ran W, Gunther SC, Pohl MO, et al MHC class II proteins mediate cross-species entry of bat influenza viruses. Nature. 2019;567(7746):109–12. 10.1038/s41586-019-0955-3 . 30787439

[10] Barclay WS. Receptor for bat influenza virus uncovers potential risk to humans. Nature. 2019;567(7746):35–6. 10.1038/d41586-019-00580-5 . 30824867

[11] Roche PA, Furuta K. The ins and outs of MHC class II-mediated antigen processing and presentation. Nat Rev Immunol. 2015;15(4):203–16. 10.1038/nri3818 25720354 PMC6314495

[12] Wosen JE, Mukhopadhyay D, Macaubas C, Mellins ED. Epithelial MHC Class II Expression and Its Role in Antigen Presentation in the Gastrointestinal and Respiratory Tracts. Front Immunol. 2018;9:2144 10.3389/fimmu.2018.02144 30319613 PMC6167424

[13] Li Q, Sun X, Li Z, Liu Y, Vavricka CJ, Qi J, et al Structural and functional characterization of neuraminidase-like molecule N10 derived from bat influenza A virus. Proc Natl Acad Sci U S A. 2012;109(46):18897–902. 10.1073/pnas.1211037109 23012237 PMC3503196

[14] Zhu X, Yang H, Guo Z, Yu W, Carney PJ, Li Y, et al Crystal structures of two subtype N10 neuraminidase-like proteins from bat influenza A viruses reveal a diverged putative active site. Proc Natl Acad Sci U S A. 2012;109(46):18903–8. 10.1073/pnas.1212579109 23012478 PMC3503178

[15] Ciminski K, Ran W, Gorka M, Lee J, Malmlov A, Schinkothe J, et al Bat influenza viruses transmit among bats but are poorly adapted to non-bat species. Nat Microbiol. 2019 10.1038/s41564-019-0556-9 . 31527796 PMC7758811

[16] Johnson KEE, Song T, Greenbaum B, Ghedin E. Getting the flu: 5 key facts about influenza virus evolution. PLoS Pathog. 2017;13(8):e1006450 10.1371/journal.ppat.1006450 28837690 PMC5600439

[17] Smith GJ, Vijaykrishna D, Bahl J, Lycett SJ, Worobey M, Pybus OG, et al Origins and evolutionary genomics of the 2009 swine-origin H1N1 influenza A epidemic. Nature. 2009;459(7250):1122–5. 10.1038/nature08182 . 19516283

[18] Long JS, Mistry B, Haslam SM, Barclay WS. Host and viral determinants of influenza A virus species specificity. Nat Rev Microbiol. 2019;17(2):67–81. 10.1038/s41579-018-0115-z . 30487536

[19] Moreira EA, Weber A, Bolte H, Kolesnikova L, Giese S, Lakdawala S, et al A conserved influenza A virus nucleoprotein code controls specific viral genome packaging. Nat Commun. 2016;7:12861 10.1038/ncomms12861 27650413 PMC5035998

[20] Juozapaitis M, Aguiar Moreira E, Mena I, Giese S, Riegger D, Pohlmann A, et al An infectious bat-derived chimeric influenza virus harbouring the entry machinery of an influenza A virus. Nat Commun. 2014;5:4448 10.1038/ncomms5448 . 25055345 PMC5533278

[21] Dadonaite B, Gilbertson B, Knight ML, Trifkovic S, Rockman S, Laederach A, et al The structure of the influenza A virus genome. Nat Microbiol. 2019;4(11):1781–9. 10.1038/s41564-019-0513-7 . 31332385 PMC7191640

[22] Bolte H, Rosu ME, Hagelauer E, Garcia-Sastre A, Schwemmle M. Packaging of the Influenza Virus Genome Is Governed by a Plastic Network of RNA- and Nucleoprotein-Mediated Interactions. J Virol. 2019;93(4). 10.1128/JVI.01861-18 30463968 PMC6363987

[23] Hershberg RM, Cho DH, Youakim A, Bradley MB, Lee JS, Framson PE, et al Highly polarized HLA class II antigen processing and presentation by human intestinal epithelial cells. J Clin Invest. 1998;102(4):792–803. 10.1172/JCI3201 PubMed Central PMCID: PMC508942. 9710448 PMC508942

[24] Moreira EA, Locher S, Kolesnikova L, Bolte H, Aydillo T, Garcia-Sastre A, et al Synthetically derived bat influenza A-like viruses reveal a cell type- but not species-specific tropism. Proc Natl Acad Sci U S A. 2016;113(45):12797–802. 10.1073/pnas.1608821113 27791106 PMC5111703

[25] Corrales-Aguilar E, Schwemmle M, editors. Bats and Viruses: Current Research and Future Trends. Poole, UK: Caister Academic Press; 2020.

[26] Maher JA, DeStefano J. The ferret: an animal model to study influenza virus. Lab Anim (NY). 2004;33(9):50–3. 10.1038/laban1004-50 . 15457202

[27] Ciminski K, Pulvermüller J, Adam J, Schwemmle M. Human MxA is a potent interspecies barrier for the novel bat-derived influenza A-like virus H18N11. Emerg Microbes Infect. 2019;8(1):556–63. 10.1080/22221751.2019.1599301 30945621 PMC6455144

[28] Huson DH, Bryant D. Application of phylogenetic networks in evolutionary studies. Mol Biol Evol. 2006;23(2):254–67. 10.1093/molbev/msj030 . 16221896

