# Supplementary figures and images for: Bats reveal the true power of influenza A virus adaptability
Source: PLoS Pathog. 2020 Apr 16;16(4):e1008384. doi: 10.1371/journal.ppat.1008384 (PMC7161946; doi:10.1371/journal.ppat.1008384)

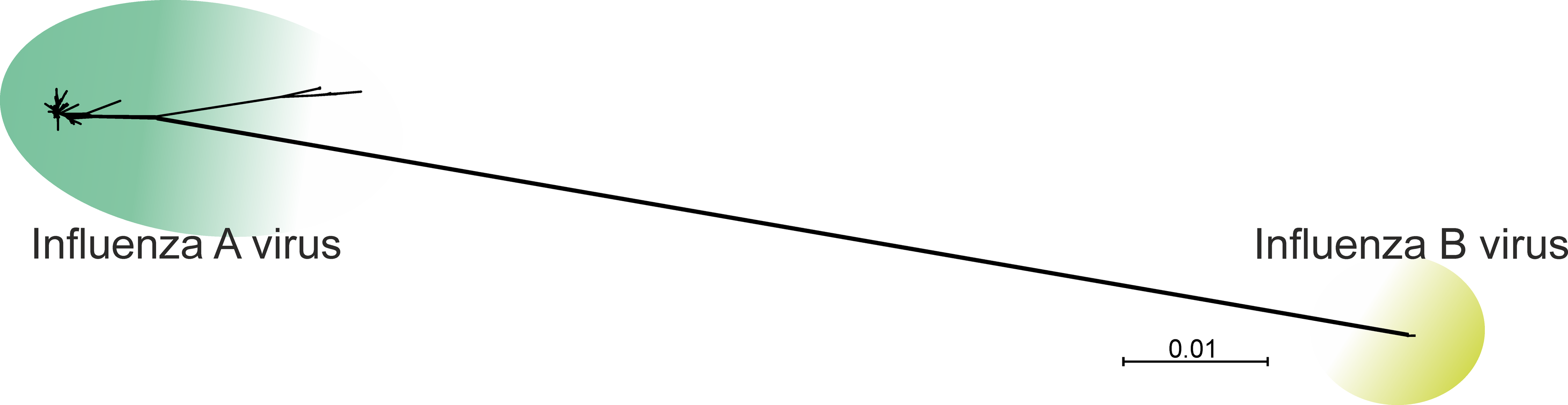

Supplement: S1 Fig — The presented phylogenetic network is based on the segments PB2, PB1, PA, NP, M, and NS from 110 representative IAVs (highlighted in green) and six influenza B viruses (highlighted in yellow). A more detailed close-up zoom of IAVs is presented in the main text (Fig 1A), and methods are described in detail in S1 Technical Appendix. IAV, influenza A virus; NP, nucleoprotein. (TIFF) [file ppat.1008384.s001.tiff]
