# Supplementary material for: Bats reveal the true power of influenza A virus adaptability
Source: PLoS Pathog. 2020 Apr 16;16(4):e1008384. doi: 10.1371/journal.ppat.1008384 (PMC7161946; doi:10.1371/journal.ppat.1008384)
Supplement: S1 Technical Appendix — (DOCX) [file ppat.1008384.s002.docx]

**Technical Appendix for**

**Bats reveal the true power of influenza A virus adaptability**

K. Ciminski, F. Pfaff, M. Beer and M. Schwemmle

**Phylogenetic Analysis**

For the phylogenetic analysis provided in Fig 1A and Fig S1 a representative set of 110 influenza A viruses (IAV) and six influenza B viruses was used. The set is based on Tong *et al.* 2013 [1] and represents all currently known IAV hemagglutinin and neuraminidase subtypes from a wide time-scale. For segments PB2, PB1, PA, NP, M and NS an nucleotide alignment was created using MAFFT (version 7.427) and a phylogenetic tree was reconstructed using IQ-TREE (version 1.6.10) [2] with options for optimal model selection [3] and 50,000 ultra-fast bootstrap replicates [4]. The resulting trees were then combined into a SuperNetwork (Z-closure super-network) using SplitsTree software (version 4.14.8) [5] applying refined heuristics, mean edge weights (weighted for tree sizes), the ZRule and 100 runs.

The alignment for PB1 was further used for a time-measured phylogeny (Fig 1B) using BEAST package (version 1.10.5) [6] based on the tip dates of each strain. Model selection of IQ-TREE suggested that the substitution model GTR with estimated base frequencies, four gamma categories and invariant sites (GTR+F+I+G4) was appropriate. An uncorrelated relaxed clock with lognormal distribution was chosen and the tree prior was set to Coalescent Skyline [7]. The optimal tree prior was chosen according to the marginal likelihood estimation using path sampling / stepping stone sampling. For a chain length of 25 million the parameters were logged every 1000^th^ and the logs were inspected using Tracer. Four independent runs were then combined using LogCombiner with a burn-in of each 8% and a maximum clade credibility tree with median heights was calculated and visualized in FigTree software (version 1.4.4).

A similar approach was chosen for an alignment of selected hemagglutinin subtype 9 (H9) (Fig 1C) sequences. In summary, all available complete nucleotide sequences for H9 from Africa, Europe and North America (n=373) were downloaded from the Influenza Virus Database https://www.ncbi.nlm.nih.gov/genomes/FLU and supplemented with the HA of A/Bat/Egypt/381OP/2017 (H9) and representative H8 and H12 sequences as outgroup.

**References**

1. Tong S, Zhu X, Li Y, Shi M, Zhang J, Bourgeois M, et al. New world bats harbor diverse influenza A viruses. PLoS Pathog. 2013;9(10):e1003657. Epub 2013/10/17. doi: 10.1371/journal.ppat.1003657. PubMed PMID: 24130481; PubMed Central PMCID: PMC3794996.

2. Nguyen LT, Schmidt HA, von Haeseler A, Minh BQ. IQ-TREE: a fast and effective stochastic algorithm for estimating maximum-likelihood phylogenies. Mol Biol Evol. 2015;32(1):268-74. Epub 2014/11/06. doi: 10.1093/molbev/msu300. PubMed PMID: 25371430; PubMed Central PMCID: PMC4271533.

3. Kalyaanamoorthy S, Minh BQ, Wong TKF, von Haeseler A, Jermiin LS. ModelFinder: fast model selection for accurate phylogenetic estimates. Nat Methods. 2017;14(6):587-9. Epub 2017/05/10. doi: 10.1038/nmeth.4285. PubMed PMID: 28481363; PubMed Central PMCID: PMC5453245.

4. Hoang DT, Chernomor O, von Haeseler A, Minh BQ, Vinh LS. UFBoot2: Improving the Ultrafast Bootstrap Approximation. Mol Biol Evol. 2018;35(2):518-22. Epub 2017/10/28. doi: 10.1093/molbev/msx281. PubMed PMID: 29077904; PubMed Central PMCID: PMC5850222.

5. Huson DH, Bryant D. Application of phylogenetic networks in evolutionary studies. Mol Biol Evol. 2006;23(2):254-67. Epub 2005/10/14. doi: 10.1093/molbev/msj030. PubMed PMID: 16221896.

6. Suchard MA, Lemey P, Baele G, Ayres DL, Drummond AJ, Rambaut A. Bayesian phylogenetic and phylodynamic data integration using BEAST 1.10. Virus Evol. 2018;4(1):vey016. Epub 2018/06/27. doi: 10.1093/ve/vey016. PubMed PMID: 29942656; PubMed Central PMCID: PMC6007674.

7. Gill MS, Lemey P, Faria NR, Rambaut A, Shapiro B, Suchard MA. Improving Bayesian population dynamics inference: a coalescent-based model for multiple loci. Mol Biol Evol. 2013;30(3):713-24. Epub 2012/11/28. doi: 10.1093/molbev/mss265. PubMed PMID: 23180580; PubMed Central PMCID: PMC3563973.
